# Polyvinyl Chloride and Polypropylene Model Nanoplastics Exhibit Distinct Interaction Patterns and Cellular Responses in Human Umbilical Vein Endothelial (HUVECs) Cells

**DOI:** 10.3390/toxics14090750

**Published:** 2026-08-26

**Authors:** Sara Bozzer, Cristina Tufoni, Murielle Salomé, Alessandra Gianoncelli, Clement Holé, Hiram Castillo-Michel, Giuseppe Ricci, Lorella Pascolo

**Affiliations:** 1 Institute for Maternal and Child Health, IRCCS Burlo Garofolo, 34137 Trieste, Italy; cristina.tufoni@burlo.trieste.it (C.T.); giuseppe.ricci@burlo.trieste.it (G.R.); 2 European Synchrotron Radiation Facility (ESRF), Beamline ID21, The European Synchrotron, 38043 Grenoble, France; salome@esrf.fr (M.S.); clement.hole@esrf.fr (C.H.); hiram.castillo_michel@esrf.fr (H.C.-M.); 3 Elettra Sincrotrone Trieste, Strada Statale 14-km 163,5 in AREA Science Park, 34149 Trieste, Italy; alessandra.gianoncelli@elettra.eu; 4 Department of Medical, Surgical and Health Sciences, University of Trieste, 34149 Trieste, Italy

**Keywords:** micro- and nanoplastics, polypropylene, polyvinyl chloride, HUVECs, endothelial toxicity, synchrotron X-ray fluorescence, LC3B-positive vesicular structures, membrane integrity, prenatal health

## Abstract

Micro- and nanoplastics (MNPs) are increasingly detected in human tissues, yet their polymer-specific effects on endothelial cells remain poorly understood, particularly at the placental and fetal level. We compared cadmium selenide quantum dot-labelled polypropylene (PP) and polyvinyl chloride (PVC) model nanoplastics (NPs) in human umbilical vein endothelial cells (HUVECs) using particle characterization, MTT assays, flow cytometry, confocal microscopy, apoptosis analysis, and synchrotron nano-X-ray fluorescence imaging. PP nanoplastics caused an early reduction in metabolic activity, showed the strongest cell-associated fluorescence, and produced the greatest increase in membrane permeability and late apoptotic/necrotic populations. PVC nanoplastics displayed a more punctate distribution with greater overlap with membrane-associated regions and induced a stronger increase in LC3B-positive vesicular structures. Nano-XRF detected Cd-enriched signals associated with the labelled particles and a polymer-specific Cd–Cl spatial association in PVC-exposed cells. Sulfur mapping further revealed localized sulfur-poor regions along the cell periphery in exposed cells, suggesting localized remodeling of peripheral membranes. These findings indicate that PP and PVC NPs interact differently with endothelial cells and elicit distinct structural and functional responses. Polymer composition should therefore be considered when assessing the vascular and prenatal effects of MNP exposure.

## 1. Introduction

Micro- and nanoplastics (MNPs) are emerging environmental contaminants that have been increasingly detected in human tissues and biological fluids, raising concerns regarding their potential impact on human health [1,2]. Recent studies have reported the presence of MNPs in blood, placenta, breast milk, and other human biological matrices, indicating that human exposure is widespread and may occur throughout sensitive developmental stages [3]. While the toxicological consequences of exposure to MNPs remain incompletely understood, growing evidence suggests that these particles can interact with cellular systems and induce oxidative stress, inflammation, metabolic alterations, and impaired cellular function [4,5,6].

Among the potential cellular targets of MNP exposure, endothelial cells are of particular interest [7]. The endothelium regulates vascular permeability, nutrient exchange, inflammatory signaling, and barrier integrity throughout the human body. These functions are especially relevant during pregnancy, where endothelial cells contribute to the maintenance of placental vascular networks and maternal–fetal exchange processes [8]. Considering the increasing evidence of MNPs in placental tissues and maternal circulation, understanding how these particles interact with endothelial cells may provide important mechanistic insights into potential effects occurring at maternal–fetal interfaces [9,10,11].

In particular, little is known at the fetal level about how different polymer compositions influence particle localization, membrane interactions, nanoscale structural alterations, and downstream cellular responses [4]. Importantly, the physicochemical properties of nanoplastics, including polymer composition, surface characteristics, and environmental ageing, are expected to influence their biological behavior, suggesting that different polymers may trigger distinct cellular responses [12,13,14,15].

Among environmentally relevant polymers, PP and PVC differ substantially in their chemical composition and surface-related properties [16]. PP is a non-polar polyolefin composed of a hydrocarbon backbone, whereas PVC contains polar C–Cl bonds [17,18]. These compositional differences may influence particle wettability, dispersion, interactions with biological membranes, and the adsorption of biomolecules. Experimental and computational studies indicate that nanoplastics interact with proteins, peptides and lipid membranes through polymer- and surface-dependent mechanisms, and that biological coronas can form on both PP and PVC nanoplastics [19]. However, particle hydrophobicity and biological identity are also influenced by particle preparation, surface ageing, additives and the surrounding medium. Moreover, the presence of chlorine in PVC does not itself demonstrate chloride release, which requires dedicated chemical-speciation or leaching measurements.

Most mechanistic in vitro investigations of nanoplastic toxicity have employed polystyrene particles, whereas PP and PVC remain comparatively underrepresented despite their widespread environmental occurrence. Direct comparisons of PP and PVC nanoplastics in endothelial cells are particularly limited, and their polymer-dependent cellular distribution has rarely been investigated using synchrotron-based elemental imaging at subcellular spatial resolution.

Addressing these questions requires analytical approaches capable of combining biological characterization with high-resolution particle detection. Conventional fluorescence microscopy provides valuable information on particle-associated signals but may be limited by optical resolution and potential interference from fluorescent background signals [20,21]. In contrast, synchrotron-based nano-X-ray fluorescence microscopy (nXRF) enables direct elemental detection at the nanoscale, allowing the element-specific detection and spatial localization of metal-labelled nanoplastics independently of fluorescence intensity and providing complementary information on the elemental composition of the surrounding cellular environment [22,23]. Furthermore, nXRF provides simultaneous information on endogenous elemental distributions, enabling the investigation of particle localization together with nanoscale alterations of cellular elemental organization [24].

In the present study, we investigated the interactions of cadmium selenide quantum dot-labelled (CdSe-QDs) PP and PVC model NPs [25], already employed in other in vitro studies [21,22,26,27] with human umbilical vein endothelial cells (HUVECs). By integrating metabolic activity assays, flow cytometry, confocal microscopy, LC3B immunofluorescence and synchrotron-based nXRF imaging, we aimed to determine whether these environmentally relevant polymers exhibit distinct interaction patterns and cellular responses in endothelial cells. We hypothesized that differences in polymer composition and associated physicochemical properties would determine distinct patterns of interaction between PP and PVC model nanoplastics and HUVECs, resulting in different cell-associated distributions and cellular responses.

## 2. Materials and Methods

### 2.1. Production of Model Polymeric Nanoplastics

Cadmium selenide quantum dot-labelled polypropylene (PP) and polyvinyl chloride (PVC) nanoplastics (NPs) were produced at the European Commission Joint Research Centre (JRC, Ispra, Italy) as previously described [25]. Briefly, CdSe quantum dots (QDs) were embedded within the polymer matrix during particle synthesis to generate fluorescently traceable polymeric particles suitable for multimodal imaging applications. Nevertheless, no independent free CdSe-QD or matched unlabeled-particle exposure groups were included in the present experimental design. The synthesis protocol was designed to produce polydisperse particles within an approximate physical-size range of 50–350 nm, as previously reported. In the present study, DLS was used to determine the hydrodynamic diameter of the dispersed particles, whereas TEM was used to confirm their morphology. 

### 2.2. Cell Culture

Human umbilical vein endothelial cells (HUVECs, Sigma-Aldrich, Wilmington, DE, USA (catalogue no. C-12250)/Merck KGaA, Darmstadt, Germany) were cultured in Endothelial Cell Medium (ECM; ScienCell Research Laboratories, Carlsbad, CA, USA), consisting of basal medium supplemented with 5% fetal bovine serum (FBS, Cat. #0025), 1% Endothelial Cell Growth Supplement (ECGS, Cat. #1052), and 1% penicillin/streptomycin solution (P/S, Cat. #0503), according to the manufacturer’s instructions. Cells were maintained at 37 °C in a humidified atmosphere containing 5% CO_2_ and were routinely passaged upon reaching approximately 80–90% confluence. Cells between passages 4 and 6 were used for all experiments.

### 2.3. Dynamic Light Scattering (DLS) Analysis

The hydrodynamic diameter and polydispersity index (PDI) of PP and PVC nanoplastics were determined by dynamic light scattering (DLS). Prior to analysis, particle suspensions were diluted 1:500 in ultrapure water and gently mixed to ensure homogeneous dispersion. Measurements were performed at 25 °C using a Zetasizer Lab Blue (Malvern Panalytical, Malvern, UK). Three independent measurements were acquired for each sample, and results were expressed as mean ± standard deviation (SD).

### 2.4. Transmission Electron Microscopy (TEM)

Particle morphology was evaluated by transmission electron microscopy (TEM). PP and PVC nanoplastic suspensions were diluted 1:100 in ultrapure water and deposited onto carbon-coated copper grids. After air drying at room temperature, samples were analyzed using a CM200 transmission electron microscope (Philips, Eindhoven, The Netherlands) operated at 100 kV. TEM images were acquired to assess particles and the presence of electron-dense CdSe quantum dots embedded within the polymer matrix.

### 2.5. Viability Evaluation

HUVECs were seeded at a density of 20 × 10^3^ cells/well in clear, flat-bottom 96-well tissue-culture plates and exposed to PVC or PP QDs/NPs at concentrations of 2, 20, or 50 µg/mL for 24, 48, 72, or 96 h at 37 °C. At each time point, 10 µL of MTT solution (20 mg/mL; Sigma-Aldrich) was added to each well and cells were incubated for 4 h at 37 °C. The culture medium was then removed, and the resulting formazan crystals were dissolved in 100 µL of DMSO. Absorbance was measured at 570 nm using a GloMax plate reader (Promega, Madison, WI, USA). Cell viability was expressed as a percentage relative to untreated controls. Experiments were performed in 3 independent replicates, with 3 technical replicates per condition. Untreated cells maintained under the same experimental conditions were used as the negative control. An independent cytotoxic positive control was not included.

### 2.6. Confocal Microscopy Analysis

HUVECs (20 × 10^3^ cells/well) were seeded on glass coverslips in clear, flat-bottom 24-well tissue-culture plates and exposed to PVC or PP QDs/NPs (50 μg/mL) for 24 h at 37 °C. After incubation, cells were washed with phosphate-buffered saline (PBS) and stained with Vybrant™ Fast DiD membrane dye and DAPI (Thermo Fisher Scientific, Waltham, MA, USA) according to the manufacturer’s instructions.

Images were acquired using a Zeiss LSM 900 confocal laser scanning microscope equipped with a 40× objective. DAPI, Fast DiD and CdSe-QD fluorescence were acquired sequentially using the appropriate excitation and emission settings to minimize spectral overlap.

Image analysis was performed using Fiji (ImageJ 1.54p). Mean fluorescence intensity was quantified from regions of interest (ROIs) defined around individual cells after background subtraction and using identical acquisition and analysis parameters for all experimental groups. Representative images from three independent experiments were used for qualitative analysis, whereas fluorescence quantification was performed on all analyzed images.

### 2.7. LC3B Immunofluorescence Analysis

HUVECs (20 × 10^3^ cells/well) were seeded on glass coverslips in clear, flat-bottom 24-well tissue-culture plates and exposed to PP or PVC QDs/NPs (20 and 50 μg/mL) for 24 h. Starved cells were included as a reference condition characterized by increased LC3B punctate staining. Following treatment, cells were washed with PBS and fixed in ice-cold 100% methanol for 15 min at −20 °C. After three washes with PBS, samples were blocked for 1 h at room temperature in blocking buffer (PBS containing 5% normal serum and 0.3% Triton X-100).

Cells were incubated overnight at 4 °C with rabbit anti-LC3B primary antibody (Cell Signaling Technology, Danvers MA, USA, Cat. N° 3868S, 1:2000) diluted in antibody dilution buffer (PBS containing 1% BSA and 0.3% Triton X-100). After three PBS washes, samples were incubated for 1 h at room temperature in the dark with Alexa Fluor^®^ 488-conjugated goat anti-rabbit IgG (H + L) secondary antibody (SouthernBiotech, Birmingham, AL, USA, Cat. N° 4030-30, 1:500). F-actin was stained with Alexa Fluor™ 647 Phalloidin (Invitrogen™, Thermo Fisher Scientific, Cat. N° A22287, 1:25), and nuclei were counterstained with DAPI. Images were acquired using a Zeiss LSM 900 confocal laser scanning microscope under identical acquisition settings for all experimental conditions.

Image analysis was performed using Fiji (ImageJ 1.54p). Mean LC3B fluorescence intensity was quantified after background subtraction using identical analysis parameters for all samples. Fluorescence intensity values were normalized to the corresponding cell area and expressed as mean ± SD from two independent experiments.

### 2.8. Lysosomal-Mimicking Fluorescence Quenching Assay

To evaluate the stability of the CdSe quantum dot (QD)-associated fluorescence under lysosomal-like acidic conditions, PP and PVC QDs/NPs were incubated in citrate buffer (pH 4.5) at 37 °C for 24 h. Fluorescence was assessed immediately after suspension preparation (0 h) and after 24 h of incubation using a ChemiDoc™ Imaging System (Bio-Rad Laboratories, Hercules, CA, USA). Images were acquired under identical exposure and acquisition settings for all samples. Fluorescence intensity was quantified in Fiji (ImageJ 1.54p) by measuring the integrated density of each sample after background subtraction. Values obtained after 24 h were normalized to the corresponding 0 h measurement and expressed as the percentage of the initial fluorescence. Experiments were performed in three independent replicates, and data are presented as the mean.

### 2.9. Synchrotron Nano X-Ray Fluorescence (nXRF) Microscopy

HUVECs were seeded at a density of 20,000 cells per well onto silicon nitride (Si_3_N_4_) windows (Silson Ltd., Warwickshire, UK) in clear, flat-bottom 24-well tissue-culture plates. After 24 h of cell adhesion, cultures were incubated with PP QDs/NPs or PVC QDs/NPs at a final concentration of 50 μg/mL for an additional 24 h. Following exposure, cells were washed with phosphate-buffered saline (PBS), fixed with 4% paraformaldehyde (PFA) for 15 min at room temperature, rinsed three times with PBS and three times with deionized water, and subsequently air-dried prior to imaging. nXRF microscopy was performed at the ID21 beamline of the European Synchrotron Radiation Facility (ESRF, Grenoble, France). A beam of 9.8 keV, monochromatized using a Si(111) monochromator, was focused down to 113 nm × 123 nm (H × V) using a pair of Pt-coated KB mirrors. The incoming beam intensity of 1.25 × 10^11^ photons/s was recorded using a photodiode upstream of the sample. The nXRF signal was detected by two five-element silicon drift detectors (500 mm^2^ active area in total, Mirion, Atlanta, GA, USA). The summed XRF spectra from these detectors were fitted using a dedicated ewoks40 workflow and the PyMCA library [28], and were calibrated against the signal derived from an AXO-standard (RF17-200-s4218-58). XRF maps were plotted using the XRFitVis web application [29].

For quantitative elemental analysis, elemental areal-density values were extracted from the cellular ROIs analysed by nXRF. Data obtained from untreated HUVECs and cells exposed to PVC QDs/NPs or PP QDs/NPs were compared statistically. The analysis included 3 untreated, 5 PVC QDs/NP-exposed and 6 PP QDs/NP-exposed cellular ROIs. Elemental areal-density values are expressed as ng/mm^2^.

Sulfur maps were processed in Fiji (ImageJ 1.54p) using a standardized segmentation workflow. Images were background-subtracted, and sulfur-poor regions within the cellular boundary were identified using the “Analyze Particles” function. Segmented features were grouped into size classes and displayed using the same color for regions belonging to the same size class. The same processing parameters were applied to all experimental conditions.

### 2.10. Flow Cytometry Analysis of Particle-Associated Fluorescence

HUVECs (250 × 10^3^ cells/well in clear, flat-bottom 24-well tissue-culture plates) were exposed to PP or PVC QDs/NPs (50 μg/mL) for 24 h. Following treatment, cells were detached, washed, and resuspended in PBS. Flow cytometric analysis was performed using a BD FACSMelody™ Cell Sorter (Becton, Dickinson and Company, Franklin Lakes, NJ, USA). A total of 10,000 events were acquired for each sample. Cell-associated fluorescence arising from CdSe quantum dots and side scatter (SSC) were recorded to evaluate particle association and changes in cellular complexity. Data were analyzed using FlowJo software (v.9).

### 2.11. Flow Cytometric Analysis of Apoptosis and Membrane Integrity

HUVECs (250 × 10^3^ cells/well in clear, flat-bottom 24-well tissue-culture plates) were exposed to PP or PVC QDs/NPs (50 μg/mL) for 24 h at 37 °C in 1 mL of complete endothelial cell medium (ECM). Following treatment, cells were harvested, washed with PBS, and stained using the Ready Flow™ Annexin V-FITC/Propidium Iodide Apoptosis Kit (Invitrogen™, Thermo Fisher Scientific) according to the manufacturer’s instructions. Briefly, 2 drops of Ready Flow™ reagent were added per 10^6^ cells, and samples were incubated for 15 min at room temperature in the dark before flow cytometric analysis. Flow cytometry was performed using a BD FACSMelody™ Cell Sorter (Becton, Dickinson and Company, Franklin Lakes, NJ, USA). Forward scatter (FSC) and side scatter (SSC) parameters were used to exclude debris, and doublets were excluded by FSC-A/FSC-H gating. A total of 10,000 events were acquired for each sample. Data were analyzed using FlowJo software (BD Biosciences, San Jose, CA, USA). Annexin V-positive, propidium iodide (PI)-positive, and Annexin V/PI double-positive cell populations were quantified and expressed as percentages of the total acquired events.

### 2.12. Statistical Analysis

Statistical analyses were performed using GraphPad Prism 10.1.1. Data are presented as mean ± SD. Comparisons among multiple groups were performed using one-way or two-way ANOVA, as appropriate, followed by Tukey’s multiple-comparisons test. A *p*-value < 0.05 was considered statistically significant. Statistical significance is reported as follows: * *p* < 0.05, ** *p* < 0.01, *** *p* < 0.001 and **** *p* < 0.0001; ns, not significant.

## 3. Results

### 3.1. Characterization of PVC and PP CdSe-QDs/NPs

**Figure 1:**
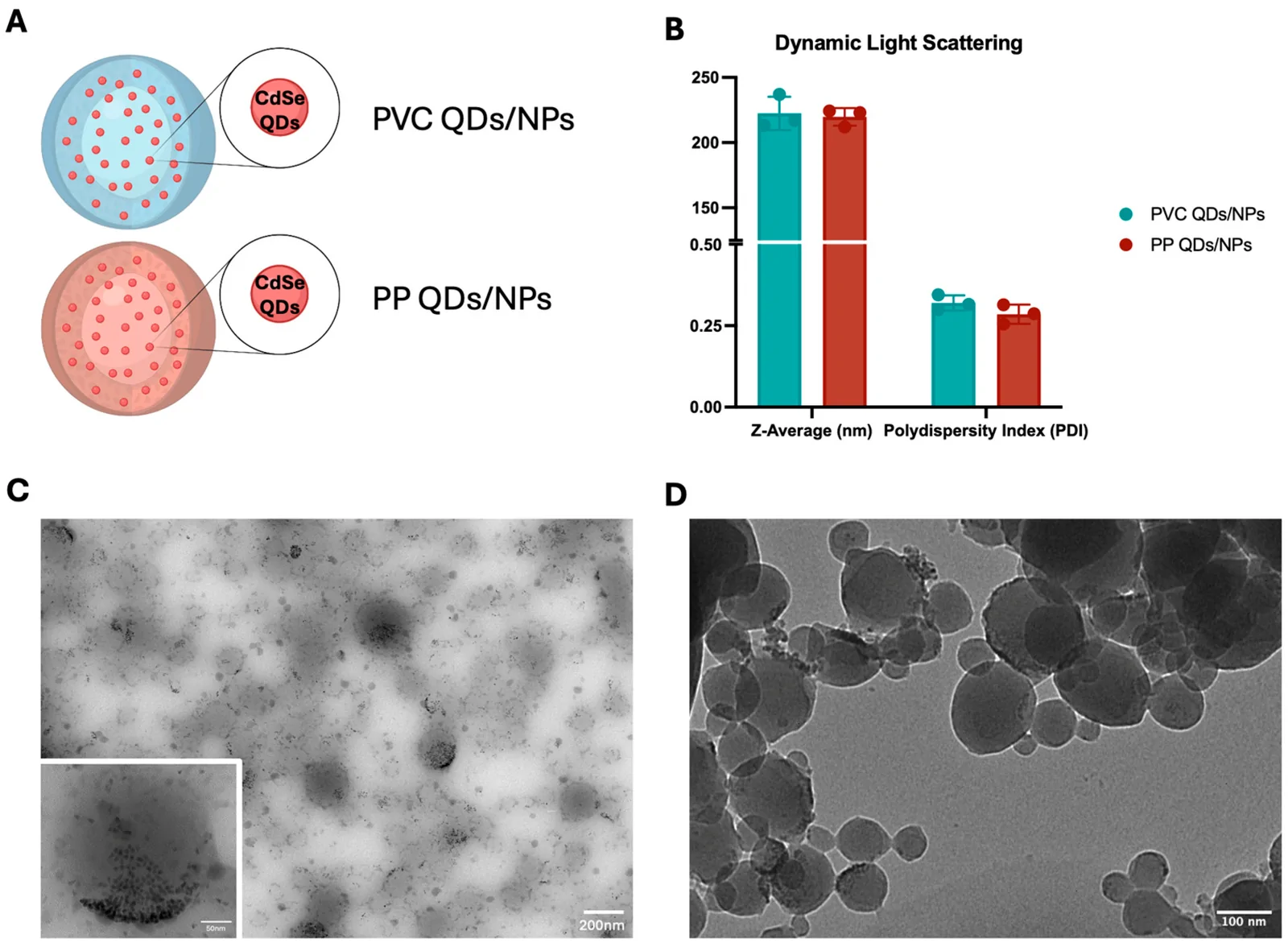
Characterization of CdSe-QD/NPs. (**A**) Schematic representation of PP and PVC NPs embedding fluorescent CdSe quantum dots (QDs; excitation ~585 nm, emission 615–650 nm). (**B**) Dynamic light scattering (DLS) analysis showing hydrodynamic diameter (Z-average) and polydispersity index (PDI) of PP QDs/NPs and PVC QDs/NPs. Data are presented as mean ± SD. (**C**,**D**) Transmission electron microscopy (TEM) images showing particle morphology and electron-dense features compatible with embedded CdSe-QDs (**C**: PP QDs/NPs; **D**: PVC QDs/NPs). Scale bars: 200 nm (**C**, inset 50 nm); 100 nm (**D**).

The quality of PP and PVC CdSe-QDs/NPs used in this study (Figure 1A) was first verified. Dynamic light scattering (DLS) analysis revealed comparable hydrodynamic diameters for the two preparations (222.4 ± 12.7 nm for PVC and 219.7 ± 6.7 nm for PP), with comparable polydispersity indices, indicating relatively homogeneous particle dispersions (Figure 1B). Transmission electron microscopy further confirmed the spherical morphology of both nanoplastic formulations (Figure 1C,D).

### 3.2. Differential Cytotoxicity and Cellular Association of PP and PVC QDs/NPs in HUVECs

**Figure 2:**
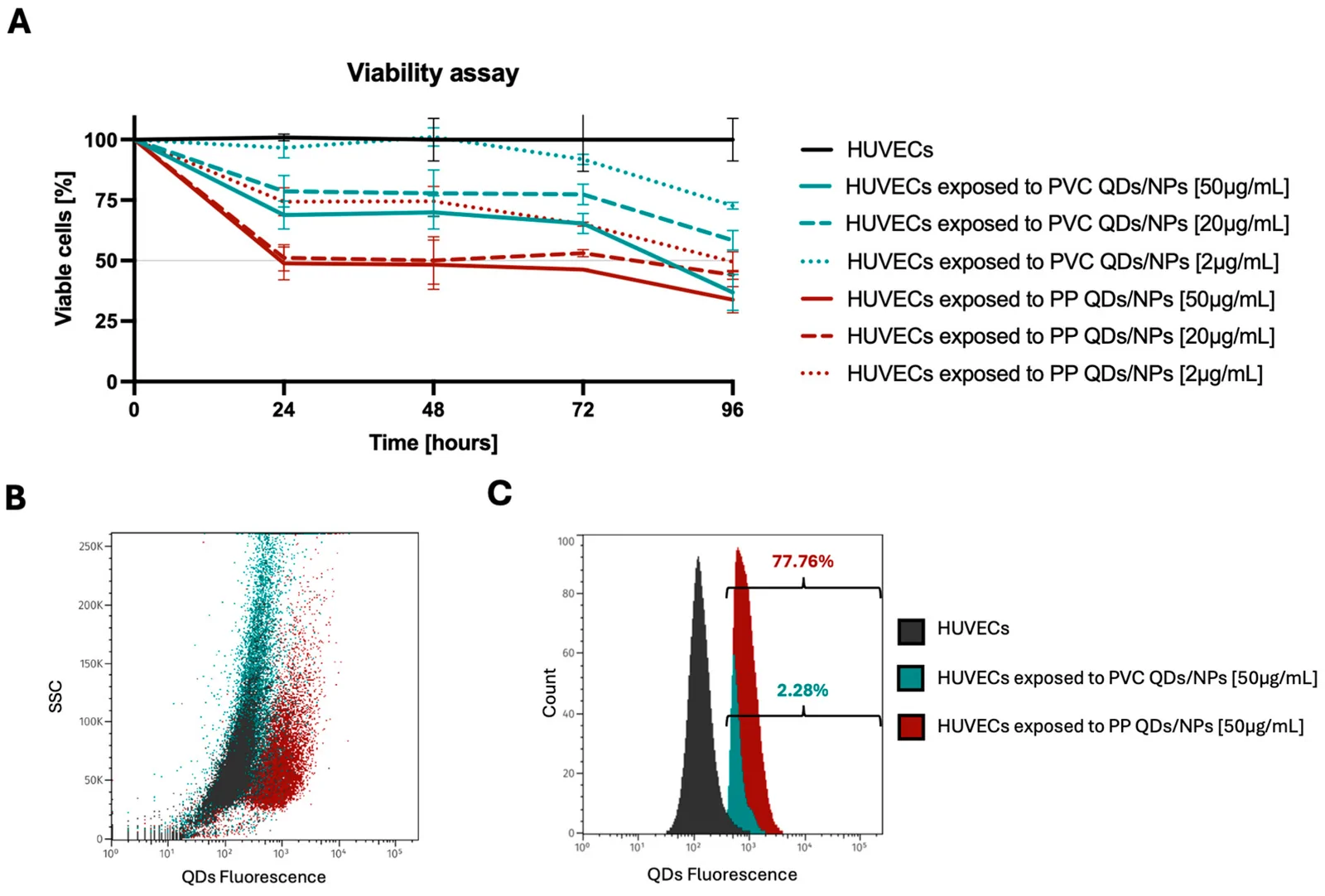
Effects of CdSe-QDs/NPs on HUVEC viability and cellular association. (**A**) Time-dependent viability of HUVECs exposed to PP QDs/NPs and PVC QDs/NPs (2, 20, and 50 µg/mL) measured by MTT assay over 96 h. Data are expressed as a percentage of viable cells relative to untreated controls (mean ± SD). (**B**) Representative flow cytometry scatter plot showing QDs fluorescence versus side scatter (SSC) in control (gray) and NP-exposed cells (light blue PVC QDs/NPs, red PP QDs/NPs). (**C**) Flow cytometry histograms of QDs mean fluorescence intensity showing particle-associated fluorescence in exposed cells, with a markedly greater fluorescence shift following PP exposure (50 µg/mL) compared to non-exposed HUVECs, indicating cellular association of QD-labelled nanoplastics.

The MTT assay was used to evaluate the cytotoxic effects of PP and PVC QDs/NPs on HUVEC viability. As shown in Figure 2A, PP QDs/NP exposure induced a concentration-dependent reduction in metabolic activity after 24 h. At 20 and 50 µg/mL, metabolic activity decreased by approximately 50%, whereas exposure to 2 µg/mL resulted in a reduction of approximately 25%. A similar reduction of approximately 25% was observed after 24 h exposure to PVC QDs/NPs at 20 and 50 µg/mL, while a decrease of approximately 50% was reached only after prolonged exposure (96 h) at the highest concentration tested.

Flow cytometry was performed to assess morphological alterations induced by both types of QD/NP exposure. After 24 h, scatter analysis revealed two populations: one displaying baseline forward and side scatter values and a second population characterized by increased side scatter, indicative of higher cellular complexity or granularity (Figure 2B).

Analysis of the QD-derived fluorescence signal confirmed particle-associated fluorescence in exposed cells (Figure 2C). PP-treated cells exhibited a pronounced fluorescence shift, with 77.76% of events displaying increased signal compared with controls, whereas PVC-treated cells showed only a minor fluorescence increase (2.28%). These results indicate a substantially higher overall cell-associated fluorescence for PP nanoplastics under the tested conditions.

### 3.3. Differential Spatial Distribution of PP and PVC QDs/NPs in HUVECs

**Figure 3:**
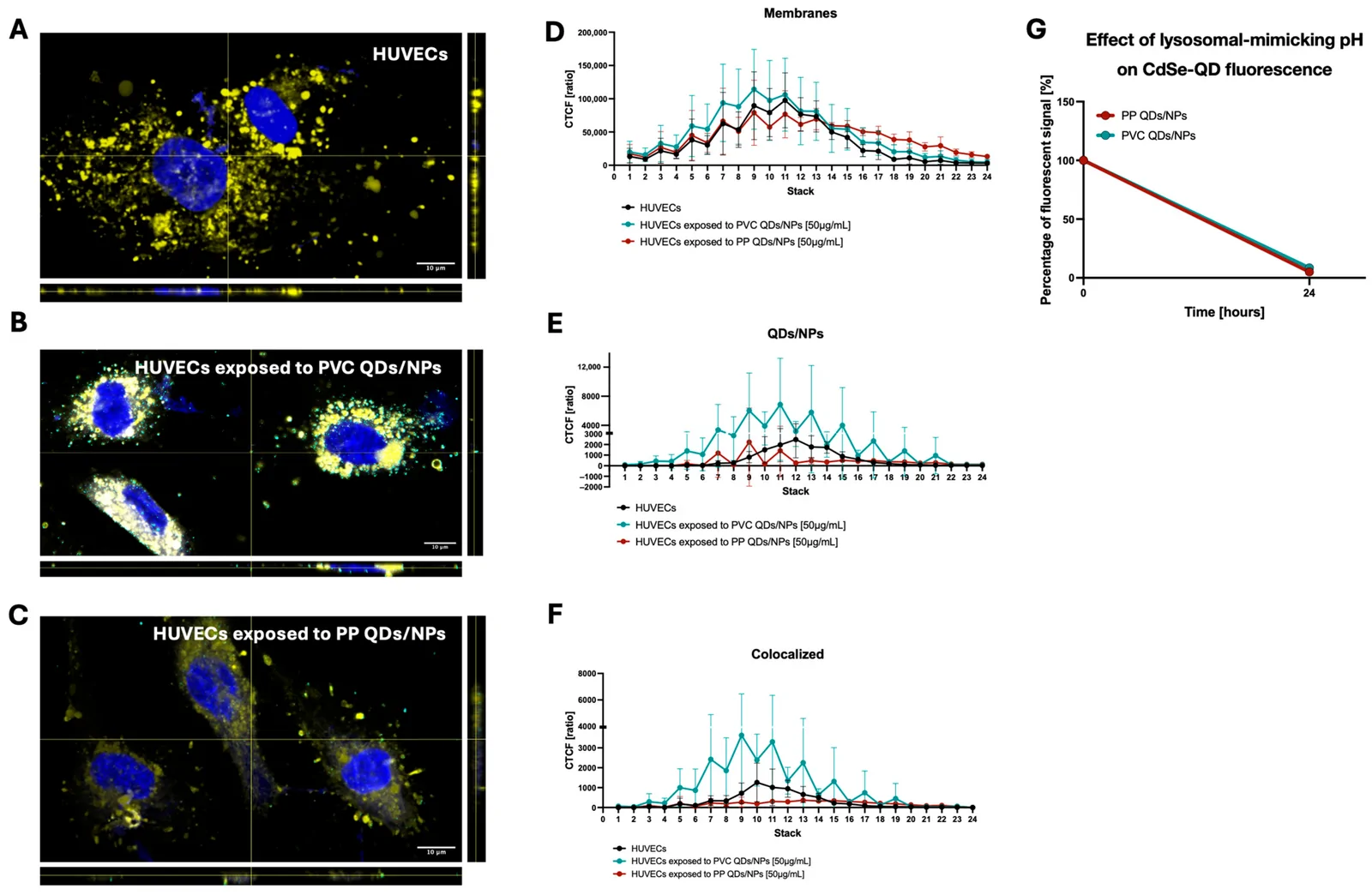
Confocal imaging reveals distinct spatial interaction patterns of PP and PVC QDs/NPs with HUVECs. (**A**,**B**) Representative confocal fluorescence images of HUVECs: (**A**) untreated control, (**B**) PVC QDs/NPs-exposed cells, and (**C**) PP QDs/NPs-exposed cells. Nuclei are stained with DAPI (blue), lipophilic cellular membranes with Fast DiD (yellow), and QDs-labelled nanoplastics (cyan). (**D**–**F**) Mean fluorescence-intensity profiles along representative cellular line scans showing the spatial distribution of (**D**) Fast DiD-associated membrane fluorescence, (**E**) QD-associated fluorescence and (**F**) spatially overlapping Fast DiD and QD-associated signals (mean ± SD). This line-profile analysis describes signal overlap but does not distinguish plasma-membrane-associated particles from particles located within intracellular membrane-bound compartments. (**G**) Stability of CdSe-QD-associated fluorescence under lysosomal-mimicking acidic conditions (pH 4.5). Fluorescence was measured at 0 and 24 h and expressed as a percentage of the initial signal.

Confocal microscopy was used to examine the spatial distribution of QDs/NPs in exposed cells. Distinct interaction patterns were observed between the two polymer types, compared to unexposed cells (Figure 3A). PVC QDs/NPs appeared as punctate cyan signals frequently localized within cellular boundaries and partially overlapping with membrane-labelled regions (Figure 3B). In contrast, PP QDs/NPs produced a stronger and more diffuse peripheral fluorescence signal (Figure 3C), consistent with a broader peripheral association.

Fluorescence intensity profiles extracted across representative cellular sections supported these different spatial patterns. While Fast DiD fluorescence remained comparable across conditions (Figure 3D), the QD-associated signal displayed spatially concentrated peaks in PVC-exposed cells (Figure 3E). The line-profile analysis also showed greater spatial overlap between the QD and Fast DiD signals in PVC-exposed cells (Figure 3F). However, because Fast DiD labels both plasma and intracellular membranes, this spatial overlap cannot distinguish surface-associated particles from particles located within intracellular membrane-bound compartments.

Flow cytometry and confocal microscopy therefore provided different but complementary observations. Flow cytometry detected substantially greater overall cell-associated fluorescence following PP exposure, whereas confocal microscopy revealed a more punctate and spatially concentrated fluorescence pattern following PVC exposure. Importantly, the subsequent quantitative nXRF analysis showed significantly higher cell-associated Cd areal density in PVC-exposed cellular ROIs than in PP-exposed and untreated cells, providing element-specific support for greater accumulation of Cd-containing signals in the analysed PVC-exposed cells. Nevertheless, because nXRF provides a two-dimensional elemental projection, this result cannot unequivocally distinguish intracellular from surface-associated particles.

Under lysosomal-mimicking acidic conditions, both particle types exhibited an almost complete loss of fluorescence after 24 h (Figure 3G), demonstrating that acidic conditions can quench the CdSe-QD-associated signal. This result identifies pH-dependent quenching as a possible contributor to the differences between the fluorescence measurements but does not establish lysosomal localization or fully explain the observed discrepancy.

### 3.4. Polymeric NPs Increase LC3B-Positive Vesicular Structures in HUVECs

Given the distinct membrane-associated interaction patterns observed in nanoplastic-exposed cells, we next investigated whether exposure to polymeric QDs/NPs altered the cellular distribution of LC3B. LC3B immunofluorescence was assessed by confocal microscopy to visualize LC3B-positive vesicular structures.

**Figure 4:**
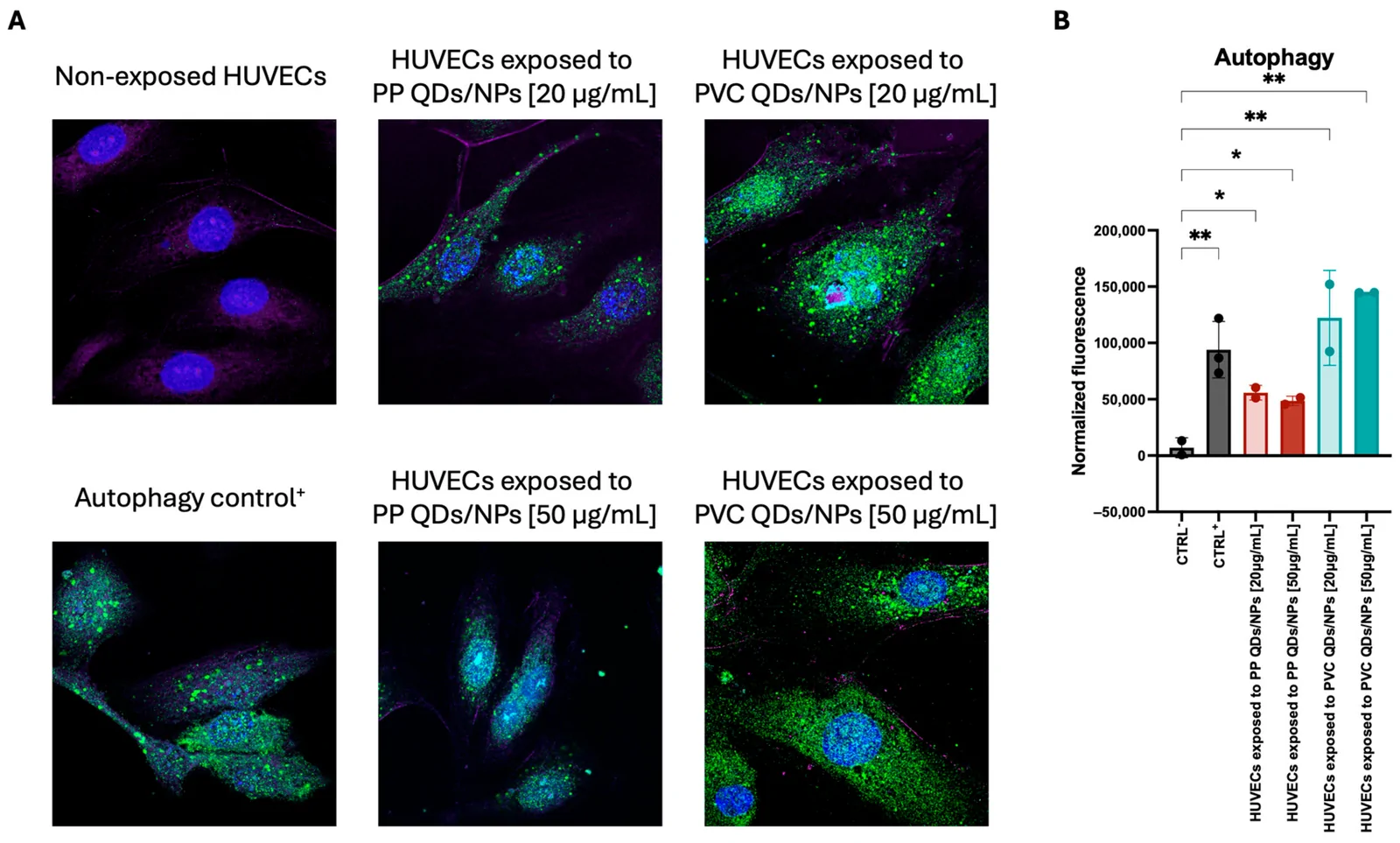
Polymeric NPs increase LC3B-associated fluorescence in HUVECs. (**A**) Representative confocal fluorescence images of HUVECs under control conditions and after exposure to PP-NPs or PVC-NPs (20 and 50 µg/mL). Cells were stained with DAPI (nuclei, blue), phalloidin (actin cytoskeleton, magenta), LC3B (autophagosome marker, green), and CdSe-QD-labelled nanoplastics (cyan). Starved cells were used as a positive control for autophagy induction. Increased LC3B puncta are observed in nanoplastic-treated cells compared with untreated controls. (**B**) Quantification of LC3B fluorescence signal (green channel) showing increased LC3-associated fluorescence in PVC-treated cells. Data are presented as normalized fluorescence (mean ± SD). Statistical significance is indicated (* *p* < 0.05, ** *p* < 0.01, *** *p* < 0.001; ns, not significant).

Representative images (Figure 4A) show the clear increase in LC3B-positive puncta (green signal) in cells exposed to polymeric NPs compared with non-exposed controls. Starved cells, used as a positive control for autophagy induction, displayed the expected strong punctate LC3B pattern. Both PP QDs/NP and PVC QDs/NP exposure resulted in more abundant LC3B-positive vesicular structures distributed throughout the cytoplasm.

Quantitative analysis of the LC3B fluorescence signal confirmed these observations (Figure 4B).

PP exposure produced a significant increase in LC3B-associated fluorescence under the tested conditions, whereas PVC exposure induced a more pronounced increase, already evident at 20 µg/mL.

These results indicate increased recruitment of LC3B to vesicular compartments following exposure to polymeric NPs. However, the identity of these compartments and their association with canonical or non-canonical autophagy-related processes cannot be determined from LC3B immunofluorescence alone.

### 3.5. Elemental Fingerprinting of Polymeric NPs by Synchrotron nXRF

**Figure 5:**
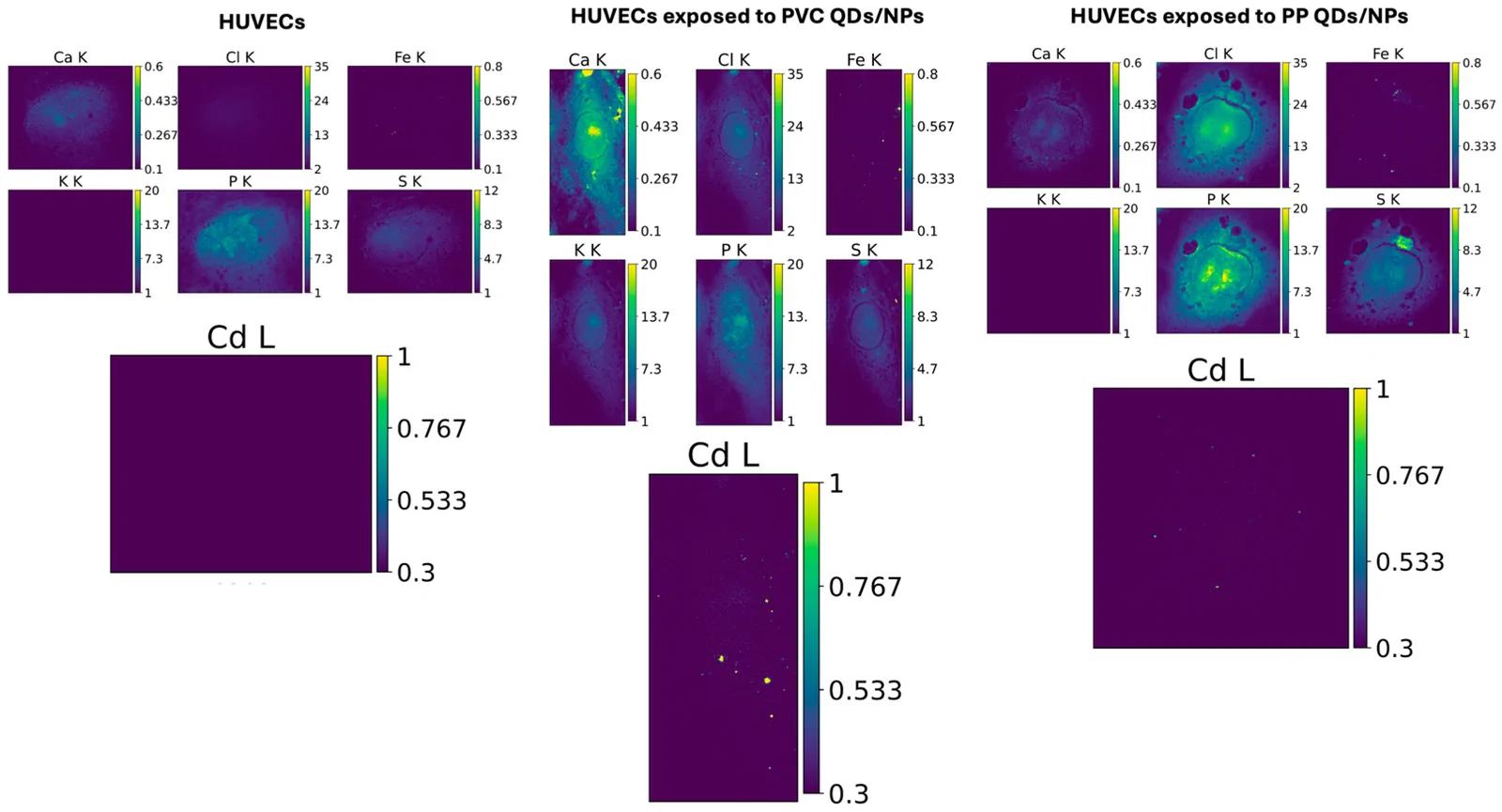
Synchrotron nXRF imaging reveals intracellular distribution of CdSe-QDs/NPs in HUVEC. Representative elemental maps obtained by synchrotron nXRF imaging showing the distribution of selected elements (Ca, Cd, Cl, S, Fe, K and P) in untreated HUVECs and cells exposed to PVC QDs/NPs or PP QDs/NPs (50 µg/mL). Measurements were performed at 9.8 keV with ~100 nm spatial resolution. Elemental signals are displayed as areal density (ng/mm^2^).

To complement the fluorescence-based observations and quantitatively assess cell-associated elemental signals, we investigated exposed cells using synchrotron-based nXRF imaging [21]. Elemental maps were acquired at 9.8 keV with approximately 100 nm spatial resolution. Representative distributions of endogenous cellular elements and Cd-labelled NPs in untreated and exposed HUVECs are shown in Figure 5.

Untreated cells displayed the expected distribution of endogenous elements, including calcium, potassium, phosphorus, sulfur and iron, whereas the cadmium signal was absent (Figure 5). In contrast, both PP- and PVC-exposed cells exhibited distinct cell-associated Cd-enriched hotspots corresponding to the CdSe-QDs embedded within the polymeric NPs. The overall distribution of endogenous elements remained preserved, allowing direct visualization of particle-associated elemental signals and cellular structures.

A clear polymer-dependent difference was observed for chlorine. In PVC QDs/NPs-treated cells, Cd-enriched hotspots frequently spatially coincided with intense punctate chlorine-rich regions, consistent with the intrinsic chlorine content of the PVC polymer. Conversely, PP-treated cells showed Cd-enriched hotspots in the absence of comparable punctate chlorine enrichment, resulting in a more diffuse chlorine distribution across the cell. These qualitative observations are consistent with polymer-dependent elemental patterns; however, endogenous cellular Cl may contribute to the detected signal.

**Figure 6:**
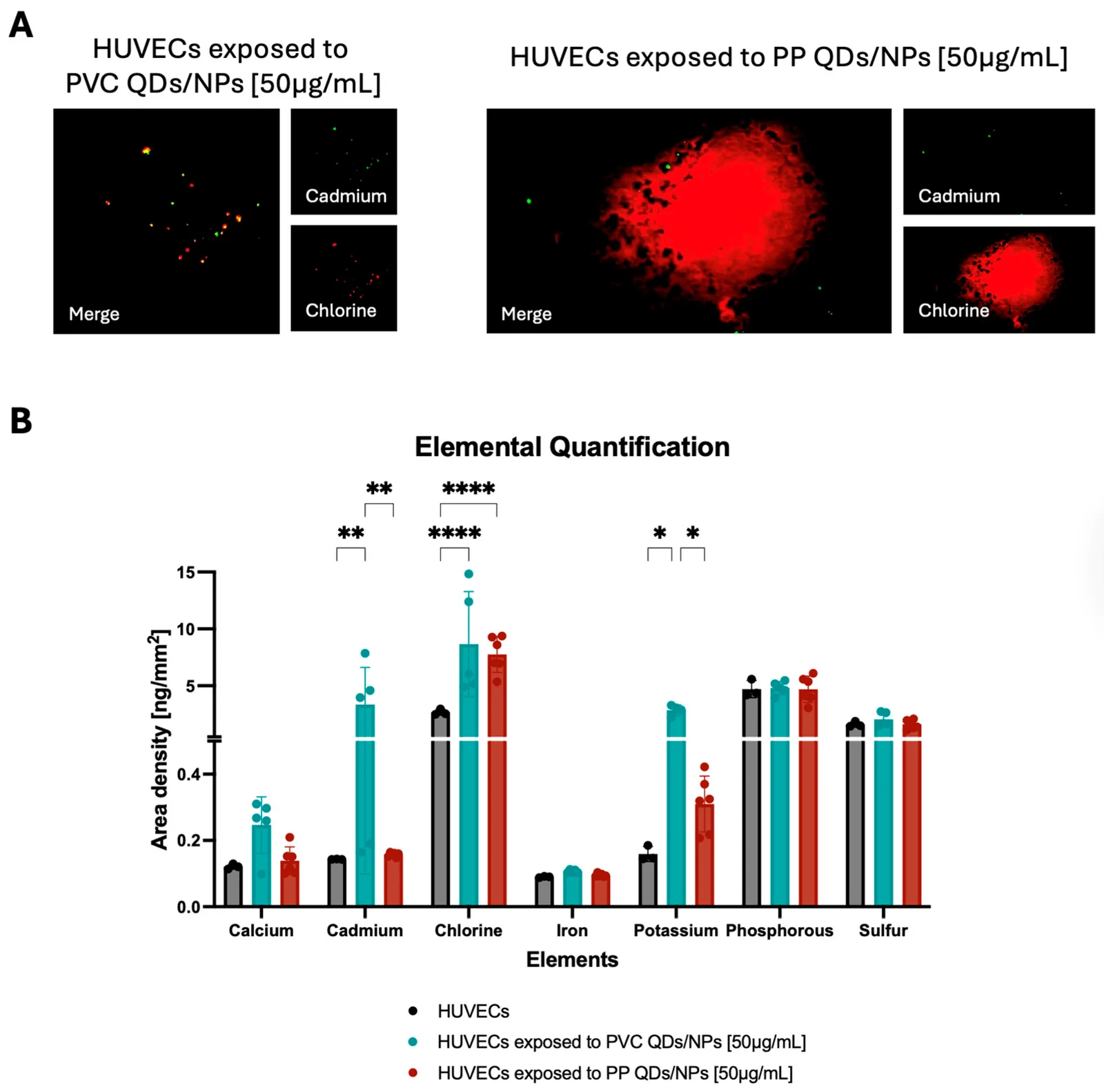
Quantitative elemental analysis of untreated and nanoplastic-exposed HUVECs. (**A**) False-color merged elemental maps showing Cd in green and Cl in red in cells exposed to PVC QDs/NPs or PP QDs/NPs. (**B**) Elemental areal density quantified within the analysed cellular ROIs and expressed as ng/mm^2^. Data are presented as mean ± SD; *n* = 3 untreated, *n* = 5 PVC QDs/NP-exposed and *n* = 6 PP QDs/NP-exposed cellular ROIs. Statistical significance: * *p* < 0.05, ** *p* < 0.01 and **** *p* < 0.0001.

To further visualize the spatial relationship between the NP tracer and the polymer-specific elemental signature (Figure 6), merged false-color maps were generated (Figure 6A), highlighting cadmium in green and chlorine in red. In PVC-treated cells, a qualitative spatial coincidence between Cd- and Cl-enriched regions was observed in representative PVC-exposed cellular maps.

Quantitative analysis of elemental areal density within the cellular ROIs revealed significant differences in Cd and Cl content among the experimental groups (Figure 6B). Cd areal density was significantly higher in PVC QDs/NP-exposed cellular ROIs than in both PP QDs/NP-exposed and untreated cells, supporting greater cell-associated accumulation of Cd-containing signals in the analysed PVC group. Potassium levels were also altered following NP exposure, whereas the remaining endogenous elements did not show significant differences under the tested conditions.

Together, these results validate the intracellular detection of polymeric NPs, in line with the results obtained by confocal microscopy. Beyond particle localization, high-resolution elemental mapping revealed localized vesicles along the cellular periphery of NP-exposed cells. Sulfur-associated signals were used here as a proxy for cellular membrane structures, although sulfur is not a specific membrane marker. These descriptive observations suggest that NP exposure may be associated with localized alterations in peripheral elemental organization.

### 3.6. High-Resolution Elemental Mapping Reveals Localized Sulfur-Poor Regions

**Figure 7:**
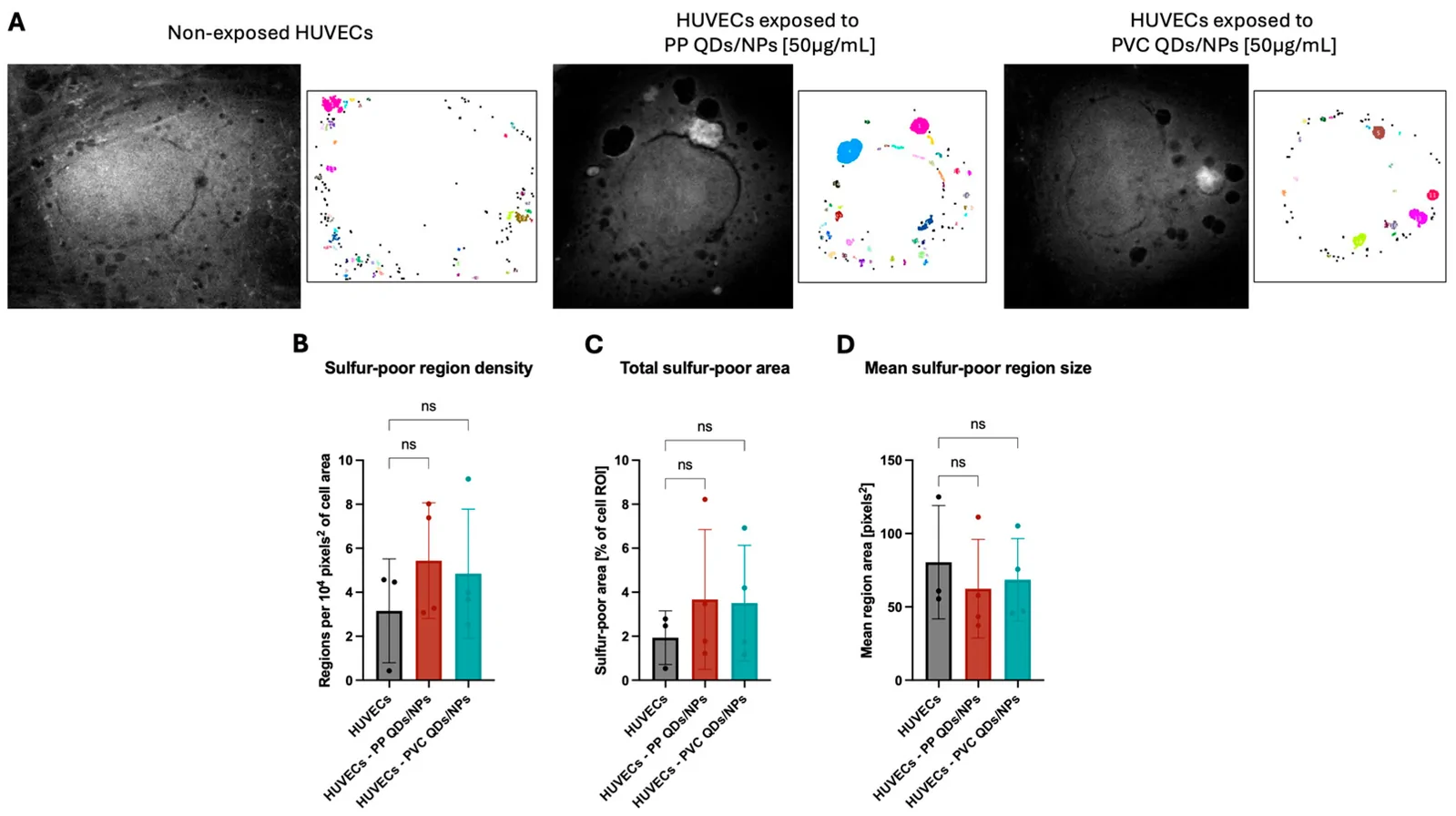
Quantitative analysis of peripheral sulfur-poor regions in untreated and nanoplastic-exposed HUVECs. (**A**) Representative sulfur nXRF maps and corresponding Fiji-based segmentations of non-exposed HUVECs and cells exposed for 24 h to PP or PVC QDs/NPs (50 µg/mL). Segmented sulfur-poor regions are color-coded according to size class. (**B**) Density of sulfur-poor regions normalized to the cellular ROI area and expressed as the number of regions per 10^4^ pixels^2^. (**C**) Total sulfur-poor area expressed as a percentage of the corresponding cellular ROI. (**D**) Mean area of individual sulfur-poor regions expressed in pixels^2^. Each point represents one independently analysed cellular ROI. Data are presented as mean ± SD; *n* = 3 for non-exposed HUVECs and *n* = 4 for each exposed group. No statistically significant differences were detected among the experimental groups under the tested conditions (ns, not significant).

The spatial distribution of sulfur (S), an element broadly associated with cellular proteins and membrane components, showed alterations in the cells correlated with NP exposure. Although sulfur is not a specific membrane marker, its distribution provides a useful proxy for membranes and peripheral structures. Representative sulfur maps are shown in Figure 7A. Fiji-based segmentation was used to quantify the density, total area and mean size of these regions (Figure 7B–D). Although the exposed groups showed numerical differences compared with untreated cells, no statistically significant differences were detected under the tested conditions. Because sulfur is not a specific membrane marker, the structural or molecular identity of these peripheral regions cannot be determined from the present nXRF data.

Untreated HUVECs displayed a largely homogeneous sulfur signal outlining the cellular shape, whereas NP-exposed cells exhibited multiple localized sulfur-poor regions along the cell periphery (Figure 7). Fiji-based segmentation was used to identify these features, which are displayed in the lower panels and color-coded according to size class. Although sulfur is not a specific membrane marker, the peripheral localization of these regions is compatible with localized remodeling of protein-rich cellular structures, including possible vesicle formation.

### 3.7. Flow Cytometric Assessment of Apoptosis and Membrane Integrity

**Figure 8:**
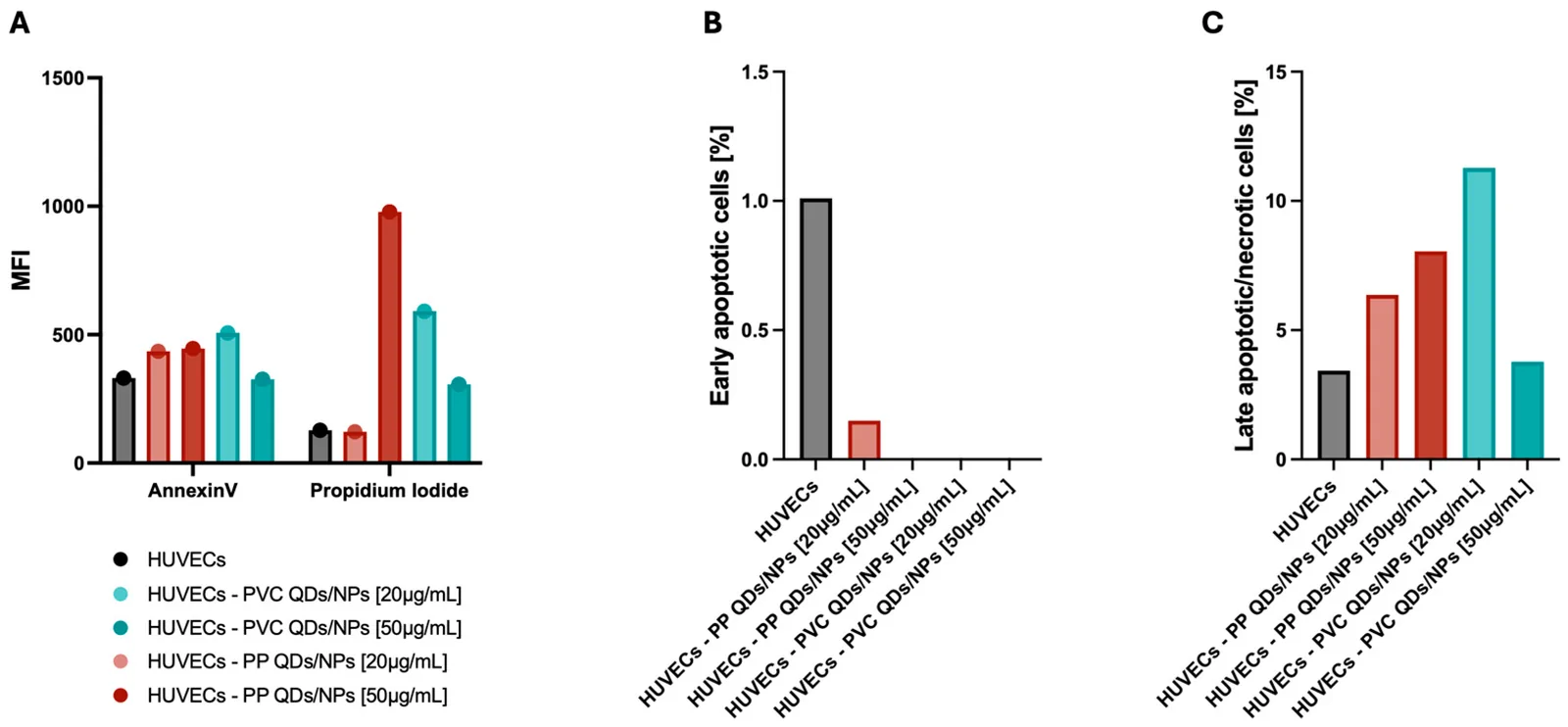
Flow-cytometric assessment of apoptosis and membrane integrity in HUVECs exposed to PP and PVC QDs/NPs. (**A**) Mean fluorescence intensity (MFI) of Annexin V and Propidium Iodide (PI) measured by flow cytometry in HUVECs exposed to PP QDs/NPs or PVC QDs/NPs (20 and 50 µg/mL). (**B**) Percentage of early apoptotic cells, defined as Annexin V^+^/PI^−^. (**C**) Percentage of late apoptotic/necrotic cells, defined as Annexin V^+^/PI^+^.

To evaluate whether the membrane sulfur-poor regions observed by nXRF imaging were associated with functional alterations of membrane integrity, apoptosis was assessed by flow cytometry using Annexin V and Propidium Iodide (PI) staining. Annexin V fluorescence reflects phosphatidylserine externalization, an early apoptotic event, whereas PI uptake indicates compromised membrane integrity. As shown in Figure 8A, NP exposure increased both signals compared with untreated cells, with the strongest PI signal observed in PP-treated cells at 50 µg/mL, consistent with increased membrane permeability. These measurements indicate alterations in phosphatidylserine exposure and membrane integrity but do not identify the molecular pathway or specific cell-death mechanism involved.

Quantification of cell populations confirmed these observations (Figure 8B). Cells exposed to polymeric NPs exhibited increased percentages of Annexin V–positive cells, indicating early apoptotic events in line with the microscopy analysis that revealed a highly advanced apoptotic activity in PVC-exposed cells (Figure 4). Notably, a marked increase in PI-positive cells was observed, particularly in cells exposed to PP QDs/NPs at 50 µg/mL, consistent with increased membrane permeability. In contrast, PP exposure at 20 µg/mL produced only a limited change in the percentage of PI-positive cells.

Overall, these findings indicate increased early apoptotic signaling and membrane permeability following NP exposure, particularly after PP exposure.

## 4. Discussion

Recent studies have reported the presence of plastic particles in human placenta and reproductive fluids, raising increasing concern about their potential impact on fertility, early development, and maternal–fetal health [30,31,32]. Within this context, endothelial cells represent a critical yet underexplored target, as they regulate vascular integrity, tissue perfusion, and barrier function in reproductive organs and at the maternal–fetal interface [33].

In this study, by integrating cytotoxicity assays, flow cytometry, confocal imaging, synchrotron nXRF, and functional membrane integrity measurements, we establish a multimodal framework to investigate NP–cell interactions across scales, from whole-cell responses to nanoscale structural alterations. Using this approach, we show that exposure of endothelial cells to polymeric NPs results in distinct and polymer-dependent interaction patterns, highlighting how physicochemical differences translate into divergent cellular responses [34].

Flow cytometry and confocal microscopy revealed different aspects of the interaction between the model nanoplastics and HUVECs. Flow cytometry showed markedly greater overall cell-associated fluorescence following PP exposure, whereas confocal microscopy showed a more punctate and spatially concentrated fluorescence pattern for PVC QDs/NPs, with greater overlap between the QD and Fast DiD signals. These observations are not directly equivalent because flow cytometry measures total event-associated fluorescence, whereas confocal microscopy provides spatial information from selected optical sections. Moreover, Fast DiD labels both plasma and intracellular membranes; therefore, its spatial overlap with the QD signal cannot unequivocally distinguish surface-associated from internalized particles.

Quantitative nXRF analysis provided complementary, fluorescence-independent evidence. Cd areal density was significantly higher in PVC QDs/NP-exposed cellular ROIs than in PP QDs/NP-exposed and untreated cells, supporting greater cell-associated accumulation of Cd-containing signals in the analysed PVC group. This elemental result is consistent with the spatially concentrated PVC-associated signals observed by confocal microscopy. Nevertheless, because nXRF produces two-dimensional elemental maps, surface-associated particles cannot be completely excluded, and the finding should not be interpreted as definitive proof of greater intracellular PVC uptake.

These distinct particle–cell interaction patterns were accompanied by different cellular responses. While PP exposure was associated with stronger membrane permeability and apoptotic signals, PVC QDs/NPs induced a more pronounced accumulation of LC3B-positive vesicular structures. The increased LC3B fluorescence and punctate pattern indicate enhanced LC3 recruitment to vesicular compartments. However, LC3B immunostaining alone cannot unambiguously identify these structures as canonical double-membrane autophagosomes [35,36]. LC3 lipidation can also occur on single-membrane endosomes, phagosomes, and lysosome-related compartments through non-canonical ATG8 conjugation pathways [37,38]. The greater spatial overlap observed for PVC QDs/NPs may therefore reflect the engagement of intracellular vesicular trafficking and endolysosomal processing, although the precise pathway remains to be determined [39,40].

To complement the fluorescence-based observations, we applied synchrotron nXRF imaging, which enabled element-specific mapping at subcellular spatial resolution [23,41]. Cd-enriched hotspots consistent with the CdSe-QD label were detected in exposed cellular ROIs. Representative PVC-exposed maps also showed spatial coincidence between Cd- and Cl-enriched regions, consistent with the co-detection of the CdSe-QD tracer and the chlorine-containing PVC polymer.

Beyond particle localization, high-resolution elemental mapping revealed localized sulfur-poor regions along the cellular periphery of NP-exposed cells. Sulfur-associated signals were used here as a proxy for protein-rich peripheral cellular structures, although sulfur is not a specific membrane marker [24]. These descriptive observations suggest that NP exposure may be associated with localized alterations in peripheral elemental organization. Further correlative imaging will be required to determine the precise structural nature of these features. 

Importantly, these structural observations were complemented by flow cytometric analysis of Annexin V and propidium iodide staining, which showed increased phosphatidylserine externalization and membrane permeability in exposed cells.

The stronger PI signal observed in PP-treated cells is consistent with a model in which surface-associated NPs may induce localized alterations in membrane integrity, leading to increased permeability and activation of cell death pathways; however, further molecular characterization (e.g., RIPK3/MLKL activation) would be required to confirm necroptosis involvement [42,43]. Together, these complementary findings support an association between nanoscale peripheral alterations and functional impairment of membrane integrity.

From a translational perspective, our findings may be relevant to perinatal and pediatric health. The detection of nanoplastics in human placenta supports the plausibility of fetal exposure, while endothelial cells are essential for placental vascular function, fetal circulation, and the maintenance of vascular barriers during development [31,32,44,45]. The polymer-dependent alterations observed in HUVECs—including changes in metabolic activity, membrane permeability, LC3B-associated vesicular responses, and peripheral sulfur organization—identify cellular processes that could be investigated as candidate mechanisms of NP-induced vascular stress [2,46]. However, the present in vitro model does not reproduce the complexity of the placental or developing vascular environment, and the tested concentrations cannot be directly extrapolated to human exposure levels. For comparison, in an exposure context, Leslie et al. reported a mean total quantifiable plastic-polymer concentration of 1.6 µg/mL in human whole blood [47], which is much lower than our tested concentrations. However, their analytical method targeted only particles ≥ 700 nm and PP was analysed but remained below the limit of quantification. Future studies should therefore evaluate these responses in primary placental endothelial cells, co-culture barrier systems, organoids, and exposure scenarios that more closely reflect conditions relevant to fetal development and early life. Such studies may help determine whether polymer composition influences vascular vulnerability during sensitive developmental windows and may support the identification of mechanistic biomarkers for environmental risk assessment in pediatric populations.

Overall, this study provides nanoscale and functional evidence that NP–cell interactions are associated with alterations in endothelial cell structure and membrane integrity. The integration of multimodal imaging and functional assays highlights the value of combining complementary techniques to investigate the biological effects of NPs. Such approaches will be essential to advance our understanding of how environmental nanomaterials interact with human tissues and to guide future studies addressing their potential impact on vascular, perinatal, and developmental health.

### Study Limitations and Future Perspectives

CdSe-QDs provide a valuable tool for tracing the biological fate of plastic polymers. Although their incorporation may introduce exogenous metal-related effects in in vitro toxicological studies, previous experiments comparing unlabeled and CdSe-QD-labeled PP and PVC nanoparticles showed that the label did not significantly increase cytotoxicity under exposure conditions comparable to those used in the present study [22]. Nevertheless, environmental plastic particles can adsorb, accumulate, and transport potentially toxic metals from their surroundings, making combined plastic–metal exposure biologically relevant, particularly in long-term exposure studies [48].

In addition, the present findings were obtained using an in vitro HUVEC monoculture. While the concentrations used in this study are relevant compared to an in vivo exposure and resulting blood concentration [47], the model does not reproduce the cellular complexity, barrier organization, hemodynamic conditions, or maternal–fetal interactions of the placental vascular environment.

Future studies should extend this work to more clinically relevant experimental models, while retaining the combination of advanced imaging and functional techniques established here and in previous studies [21,26,27,49].

## 5. Conclusions

Our findings show that CdSe-QD-labelled PP and PVC model NPs exhibit distinct interaction patterns and induce different cellular responses in HUVECs. PP QDs/NPs showed greater overall plasma membrane-associated fluorescence and were linked with an early reduction in metabolic activity, increased membrane permeability, and apoptotic responses. In contrast, PVC QDs/NPs are more efficiently internalized with a greater spatial overlap with intracellular membranes, and a stronger increase in LC3B-associated fluorescence. Synchrotron nano-XRF provided complementary elemental detection of the labelled particles, including Cd–Cl spatial association in PVC-exposed cells, and revealed localized sulfur-poor regions at the cellular boundary. More importantly, this technique confirmed the differential uptake and toxicity of the two polymers.

These findings highlight the importance of considering polymer composition when investigating the biological effects and potential developmental risks associated with NP exposure.

## Data Availability

The data presented in this study are available within the article. In accordance with the ESRF data policy, the raw synchrotron data are available at DOI: 10.15151/ESRF-ES-2146821348.
